# Examining the Effects of Raw Material and Product Prices on the Payback Time and Net Present Value for the Cumene Production Process: Simultaneous Use of SuperPro Designer and Central Composite Design Programs

**DOI:** 10.1021/acsomega.6c02060

**Published:** 2026-07-14

**Authors:** Ali Yalçın

**Affiliations:** Department of Chemical Engineering, Faculty of Engineering and Natural Sciences, 52994 Süleyman Demirel University, Isparta 32260, Turkey

## Abstract

Throughout history, adverse events that have caused global economic crises, such as war, pandemics, global warming, and climate change, have led to fluctuations in the prices of crude oil, coal, and other energy commodities. These fluctuations directly drive price volatility in various chemical products derived from crude oil distillation. This work aims to simultaneously use SuperPro Designer and central composite optimization to optimize payback time and net present value (NPV), based on raw material and product prices, in the cumene production process (CPP). The reduced cubic response surface and 2FI models explain all variations in payback time and NPV at the applied price scale, and analysis of variance for both models was significant. The *R*-squared, adjusted *R*-squared, and predicted *R*-squared values for both models were well above acceptable levels, indicating excellent generalization and confirming the models’ suitability for estimating payback time and NPV across varying price scenarios. The dual interactions between benzene–cumene and propylene–cumene prices create a synergistic effect. The results show that as raw material prices rise, the payback time increases; however, this synergistic effect reduces it as the cumene selling price rises. The total capital investment and operating costs of the CPP are 9.88 and 96.41 million USD, respectively, for a production capacity of 89,000 MT/year. Moreover, the hypothetical payback time, NPV, and cumene production cost are 2.8 years, 29.383 million USD, and 1090 USD/MT, respectively, based on the same production rate. In addition to the techno-economic analysis of the CPP, the combined use of design and optimization programs adds value to this study. It provides notable insights for investors, process engineers, and researchers.

## Graphical abstract

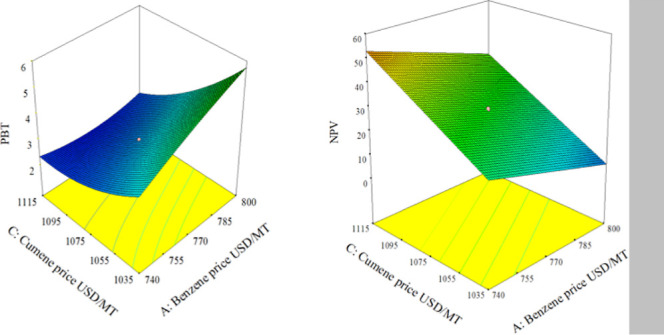

## 1 Introduction

In the past few years, the world has faced global economic crises such as food insecurity, rising gold prices, fluctuations in oil and energy prices, and drought due to various factors, which directly affect the economies of countries, such as the COVID-19 pandemic, the Russia–Ukraine war, Pakistan–India tension, the conflicts in the Middle East, the increase in U.S. customs taxes, climate change, and global warming.^1–6^ Although countries have made efforts to reduce these tensions at certain times and to minimize the effects of the COVID-19 pandemic, climate change, and global warming,^7^ similar actions may emerge in the coming years, and as a result, it can be said that the world may face a new economic crisis, potentially accompanied by drought, food, oil, and energy crises.^8,9^ These global issues cause fluctuations in the prices of energy resources, including oil, coal, and natural gas.^2,5,6^ For example, while the price of crude oil was ∼62 USD/Bbl in the last quarter of 2019 when the first COVID-19 case emerged, it fell to ∼22 USD/Bbl as the virus spread worldwide in the following days. On the other hand, with the start of the war between Ukraine and Russia in February 2022, the barrel price of crude oil rose from 97 USD to 121 USD within six months. Subsequently, the price of crude oil fell to 73 USD in the first quarter of 2023 due to a decline in oil demand and the onset of an economic recession. In the following years, a short-term conflict between Israel and Iran led to a 10% increase in the price of a barrel of crude oil.^10^ These significant fluctuations in crude oil prices impact the prices of various chemical materials derived from crude oil.^11^ Although price volatility caused by these and similar global concerns affects the production economy, production will continue as long as there is demand for the product.

Before investing in a petrochemical production facility, a techno-economic analysis (TEA) should be conducted by using a simulation program to ascertain the payback time (PBT), total capital investment (TCI), operating costs (OCs), net present value (NPV), and production costs.^12,13^ This analysis helps investors assess the profitability potential of the production process, provided that a TEA is performed that accounts for fluctuations in raw material and product prices. In that case, it enables investors to make more informed decisions as they seek to recover their invested money in the short term.^12,14^ Moreover, it enables the necessary measures to be taken to purchase raw materials at reasonable prices while accounting for potential price fluctuations during production due to global economic factors.

Cumene, also known as isopropyl benzene, is used as an essential feedstock for the synthesis of phenol, acetone, and their derivatives, which are main intermediates in the production of resins, plastics, coatings, and synthetic fibers.^15,16^ In 2024, global cumene volumes exceeded 15 million tons annually. The market was valued at about 22.4 billion USD. It is estimated to exceed 32.6 billion USD by 2034, with a compound annual growth rate of about 4.8%.^16^ It is mainly produced by the cumene production process (CPP) that yields cumene and diisopropylbenzene (DIPB) via a gas-phase reaction between benzene and propylene at high temperature and pressure.^16–18^ In the literature, the CPP has been described and analyzed using various simulation programs. In the studies evaluating the economic analysis of the CPP performed by Luyben (2010),^19^ Maity et al. (2013),^20^ Flegiel et al. (2015),^15^ Zhai et al. (2015),^21^ Pathak et al. (2011),^17^ and Norouzi et al. (2014),^22^ the TCIs were presumed to be 7.33, 8.13, 17.87, 8.22, 5.90, and 8.09 million USD, respectively. At the same time, OCs were estimated at 132.71, 140.31, 469.41, 143.24, 140.11, and 138.97 million USD, respectively. Moreover, all these studies reported a 3 year hypothetical PBT for the cumene process. This process was simulated using design programs such as CHEMCAD, Aspen Plus, and an Excel-based MOO program. In these studies, an economic analysis of the CPP was conducted using a single sales price for raw materials and products, based on a given cumene production capacity, resulting in limited economic data to evaluate the process economics.^15,21,23^ The prices of raw materials and products in this production process are directly affected by crude oil and global energy prices.^6^ Volatility in supply, demand, and crude oil prices directly affects the purchasing prices of benzene–propylene and the selling prices of cumene–DIPB for the CPP. This instability in raw material and product prices also affects the economic assessment of the CPP. The facility’s economic situation should be evaluated not only by considering the changes in raw material prices but also by accounting for product price fluctuations and the balance of supply and demand. Measures should be taken to address possible problems arising from global economic concerns and to ensure economic stability. Performing a TEA of the CPP, considering various raw materials and product pricing, would also help identify measures to mitigate economic fluctuations that may occur during the process’s operation.

SuperPro Designer (SPD) is a software tool used to simulate, evaluate, and optimize integrated batch and continuous manufacturing processes across the chemical, biochemical, and environmental industries as well as to conduct TEAs of related processes. It is a useful and user-friendly design program for simulating large-scale production processes and predicting the future feasibility of any plant.^24^ The software features a comprehensive library that includes the thermodynamic data for chemicals and mixtures. Many convenient features enable equipment sizing and costing calculations by performing mass and energy balance calculations for each piece of equipment in the production process.^24,25^ The recycle loop and tear stream option commands are enabled by the simulator to model the massive processes at various production capacities. It identifies an iteration mechanism for the stream convergence criterion within each recycling loop. The final output for all processes is derived after all iteration loops converge.^13,26^ Although SuperPro Designer performs detailed technoeconomic assessments and calculates indicators such as PBT, TCI, OCs, and NPV, it lacks an embedded multivariable optimization framework to determine their optimal values under simultaneous price variations.^27^

Central composite design (CCD) is one of the widely applied methods for optimizing experimental design parameters using response surface methodology (RSM), particularly preferred for modeling, optimizing, and understanding complex multivariable experimental procedures across various scientific, research, and engineering disciplines. Applying a one-factor-at-a-time approach, in which a variable is changed while keeping other factors constant, results in a costly, time-consuming experimental design and fails to detect interactions between variables. It is used to overcome these limitations by optimizing key variables in complex processes. The program enables the use of CCD by reducing the number of experiments required in traditional designs, thanks to its efficiency and reliability, and makes it very useful for identifying the optimal values of key variables in fields such as chemical engineering, chemistry, waste recovery, materials science, biotechnology, process optimization, and process development.^28^ CCD ascertains the relationship between independent variables and response functions by combining factorial, axial, and center points. This structure enables the improvement of second-order polynomial models without a full three-level factorial design. It allows curvature effects to be derived with high statistical resolution, thereby lowering the experimental cost.^29^ Furthermore, integrating CCD optimization programs with simulation tools such as SPD and Aspen Plus enhances their applicability to TEA of any industrial process, accounting for raw material and product price fluctuations. It facilitates the analysis and interpretation of the key economic variables in the production process.

There have been reports in recent years on the application of process simulation tools, along with advanced optimization and statistical methods, to analyze and enhance the CPP. For example, recent research has used Aspen Plus, along with auxiliary tools (e.g., MATLAB), for thermodynamic performance assessment and to propose energy efficiency enhancements based on exergy analysis.^30^ This work is concentrated on process-level optimization such as energy integration, reaction efficiency, and separation performance. Likewise, recent advances in cumene process design and optimization have centered on rigorous modeling, control schemes, and operational enhancements in an Aspen-based environment.^31^ In these works, the goal of the study is usually to improve conversion, selectivity, or energy consumption, not so much to relate process variables to economic performance metrics. While these approaches have proven useful, there have been few efforts to directly integrate process simulation outputs with techno-economic metrics within a co-optimization framework. In this regard, this paper uses SPD in conjunction with CCD to establish a direct relationship between process parameters and key economic indicators, such as NPV and PBT. Hence, the novelty of this work lies in extending the traditional simulation–optimization approach to an economy-based framework, in which the process-operating conditions are assessed simultaneously from technical and economic perspectives, accounting for fluctuations in the market environment.

This study presents a systematic attempt to integrate SuperPro Designer simulations with CCD-based optimization to evaluate the techno-economic performance driven by price. It aims to determine the effect of raw material/product price volatility on the PBT and NPV, considering various raw material purchase prices and product selling prices using SPD and CCD programs. The simulation program was run 29 times using a range of various prices for benzene (740–800 USD/MT), propylene (920–1012 USD/MT), cumene (1035–1115 USD/MT), and DIPB (2960–3256 USD/MT). The integration of input and output parameters through the simultaneous use of SPD and CCD provides practical guidance for investors, researchers, and process engineers.

## 2 Methodology

### 2.1 Cumene Production Process

SPD V9 was used to simulate the CPP and to perform its TEA, based on an 89,000 MT/year CPP. All materials, including the main product, raw materials, unreacted materials, and byproducts, required to simulate the CPP were selected from the software’s library. The CPP for cumene production includes sections such as (i) benzene pretreatment, (ii) propylene pretreatment, (iii) heating process, (iv) gas-phase reactions, (v) cooling processes, (vi) gas separation way, (vii) benzene/cumene/DIPB separation, (viii) benzene recycling line, and (ix) natural gas combustion.^23^ In the benzene pretreatment and propylene pretreatment, heating process sections, a storage tank, a multipipe heat exchanger, and two pumps are used to raise the temperatures and pressures of the raw materials and recycled/unreacted products to reaction conditions. In the gas-phase reaction section, a reactor is used to convert raw materials into products, as shown in eqs 1 and 2.^32,33^ The gas-phase reaction between benzene and propylene take places at 350 °C and 30.75 bar in the presence of a solid phosphoric acid catalyst (presumed to have 0.5 void fraction and a 1000 kg/m^3^ of bulk density for catalyst), based on the amount of the produced cumene converted into DIPB (eq 2) at the same reaction conditions.^17,19,23^ Cooling processes are used to reduce the temperature of all materials exiting the reactor and distillation columns to the required temperature. In the gas separation and benzene/cumene/DIPB separation sections, flash distillation, two distillation columns, and two storage tanks are also used to separate and purify the gases, raw materials, products, and byproducts produced by gas-phase reactions according to eqs 1 and 2. The benzene recycling line feeds all materials containing high amounts of benzene from the benzene column to the benzene storage tank.^23^ In the natural gas combustion process, the flue gas, also known as combustion gas, is used to raise the temperature of all materials fed to the reactor to the reaction temperature in the CPP, as shown in Figure 1. This gas is produced by burning natural gas with air in the combustion chamber. The gases in the natural gas mixture used for flue gas production, along with their mole fractions,^34^ were recorded as follows: 0.162% C_4_H_10_, 0.620% CO_2_, 0.919% C_2_H_6_, 97% CH_4_, 0.363% C_3_H_8_, and 0.936% N_2_, as recorded from the simulation’s database. The combustion reactions of the combustible gases (C_4_H_10_, C_2_H_6_, CH_4_, and C_3_H_8_) present in natural gas with air and the heats of reaction are illustrated in eqs 3–6.^35^ To raise the temperature of the raw materials and unreacted reaction products to the reaction temperature of 350 °C and to ensure full combustion, natural gas was burned with 30 times more air by mass (stoichiometrically ∼60% excess O_2_) as the temperature generated when natural gas is burned with oxygen is typically below 1400 °C,^36^ resulting in high-temperature flue gas. To connect the unit procedures and streamlines in sequence within the CPP, a flowsheet was formed by selecting from the software library (Figure 1).

1 $$C_{6}H_{6}+C_{3}H_{6}\to C_{9}H_{12},\Delta H=-98.6\;kJ/mol$$

2 $$C_{9}H_{12}+C_{3}H_{6}\to C_{12}H_{18},\Delta H=-27.2\;kJ/mol$$

3 $$C_{4}H_{10}+{13/2O}_{2}\to {4CO}_{2}+{5H}_{2}O,\Delta H=-49.6\;kJ/kg$$

4 $${2C}_{2}H_{6}+{7O}_{2}\to {4CO}_{2}+{6H}_{2}O,\Delta H=-52.0\;kJ/kg$$

5 $${CH}_{4}+{2O}_{2}\to {CO}_{2}+{2H}_{2}O,\Delta H=-55.6\;kJ/kg$$

6 $$C_{3}H_{8}+{5O}_{2}\to {3CO}_{2}+{4H}_{2}O,\Delta H=-50.4\;kJ/kg$$

**Figure 1:**
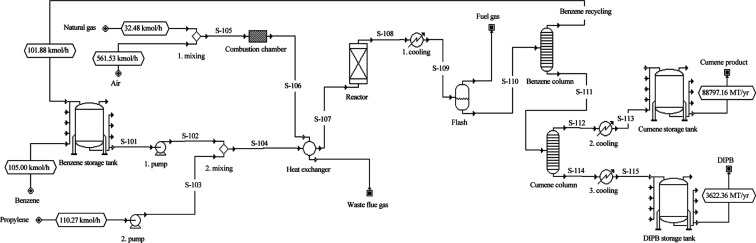
Flowsheet for CPP.

### 2.2 Experimental Design for Optimizing the Prices of Raw Materials and Products

A CCD (Stat-Ease 360R, Stat-Ease Inc., Minneapolis, USA) was used for the runs, as each run differed from the others due to interaction effects over PBT and NVP (as response-1 and response-2), the dependent variables. The experiment runs were designed with two dependent variables of PBT (years) and NVP (million USD) and four independent variables, which are purchasing price (USD/MT) of benzene (factor-1), purchasing price (USD/MT) of propylene (factor-2), selling price (USD/MT) of cumene (factor-3), and selling price (USD/MT) of DIPB (factor-4), and combined effects, to achieve optimized economic conditions in a total of 29 runs, as seen in Table 1. Annual natural gas costs were excluded from the optimization because they were low relative to the annual turnover of raw materials and products.^37^ The prices of raw materials and products, along with their corresponding levels (low and high), for the experimental design were determined from the literature prices. Based on an acceptable PBT, a four-factor, three-level (−1, 0, +1) CCD with five center-point replicates was selected to optimize the prices of raw materials and products. Appropriate models were selected based on the optimization results. The probability values (*P* < 0.05), the coefficient of determination (*R* ^2^), and the *p*-value for lack of fit were determined to assess the quality of the fitted polynomial models using the appropriate models derived from the optimization results. One-way analysis of variance (ANOVA) was used to analyze all variables, and the design levels for responses 1 and 2 were estimated using the regression model equation.^38^

**Table 1:** CCD Based on the Prices of Raw Materials/Products and Their Effect on PBT and NPV

	independent variable				dependent variable	
	purchasing price of raw materials		the selling price of products		PBT	NPV
runs	factor-1 (USD/MT)	factor-2 (USD/MT)	factor-3 (USD/MT)	factor-4 (USD/MT)	response-1 (years)	response-2 (million USD)
1	770.00	874.00	1075.00	3108.00	2.15	43.394
2	800.00	920.00	1035.00	2960.00	4.62	10.407
3	800.00	920.00	1035.00	3256.00	4.00	14.96
4	740.00	1012.00	1115.00	2960.00	2.16	43.806
5	770.00	966.00	1075.00	3108.00	2.80	29.383
6	830.00	966.00	1075.00	3108.00	4.38	12.42
7	770.00	966.00	1075.00	2812.00	3.10	24.831
8	770.00	966.00	1075.00	3404.00	2.56	33.935
9	800.00	1012.00	1115.00	3256.00	2.73	31.395
10	710.00	966.00	1075.00	3108.00	2.04	46.346
11	740.00	920.00	1035.00	3256.00	2.63	31.923
12	770.00	1058.00	1075.00	3108.00	3.99	15.371
13	740.00	920.00	1035.00	2960.00	2.89	27.37
14	800.00	1012.00	1115.00	2960.00	3.00	26.843
15	770.00	966.00	1075.00	3108.00	2.80	29.383
16	740.00	1012.00	1035.00	2960.00	4.19	13.359
17	770.00	966.00	1075.00	3108.00	2.80	29.383
18	800.00	920.00	1115.00	2960.00	2.27	40.855
19	770.00	966.00	1075.00	3108.00	2.80	29.383
20	740.00	920.00	1115.00	3256.00	1.64	62.37
21	770.00	966.00	1155.00	3108.00	1.72	59.83
22	740.00	920.00	1115.00	2960.00	1.74	57.818
23	770.00	966.00	1075.00	3108.00	2.80	29.383
24	800.00	920.00	1115.00	3256.00	2.11	45.407
25	740.00	1012.00	1035.00	3256.00	3.67	17.911
26	800.00	1012.00	1035.00	3256.00	6.87	1.958
27	740.00	1012.00	1115.00	3256.00	2.01	48.358
28	800.00	1012.00	1035.00	2960.00	8.89	–2.317
29	770.00	966.00	995.00	3108.00	7.57	–0.219

### 2.3 TEA of the CPP

TEA for simulated CPPs enables the estimation of PBT and NPV for various raw materials and product prices. The CPP was simulated at 90% capacity for 330 days. Economic analysis parameters for the entire project were estimated by using 2025 economic values. A 15 year lifetime for the cumene production facility also includes a 1 year construction and a start-up phase. The recycle loop simulation for the cumene production facility was run for 500 iterations to meet the stream convergence criterion. Depreciation over 10 years using a straight-line method and salvage value (5% of the initial cost)^39^ were calculated using the default data suggested by the SPD. It was assumed that the cumene production facility is located near the market where raw materials are purchased and products are sold; therefore, transportation costs were not included in the simulation program when calculating OCs.

OCs and TCIs for the CPPs were calculated using SPD after simulating the CPP at an 89,000 MT/year capacity, based on raw material and product prices. OC comprises total variable costs, including the purchase prices of raw materials, labor-dependent costs, facility-dependent costs, laboratory/QC/QA costs, and utilities. In contrast, TCI is the sum of the total direct cost (TDC), the total indirect cost (TIC), the contractor’s fee, and the contingency (CFC) (eq 7).^40^ Installation, salary, plant construction, equipment cost, piping, and other expenses are referred to as TDC. In contrast, design, engineering, financing, land acquisition, and due diligence are collectively known as TIC. On the other hand, the contractor’s fee is the amount the contractor charges for managing and carrying out the construction work. At the same time, contingency is a budget set aside to cover unforeseen expenses that may arise during the project.^13^ The TCI for the cumene production facility was determined based on equipment purchase costs and other costs predicted by the simulation program. Equipment purchasing costs were derived to estimate the cost of unit procedures operated for the CPP in the SupePro Designer simulator’s database. The costs of all equipment and raw materials were calculated based on the purchasing prices, while those of the main product and byproduct were set at selling prices.

A TEA of the CPP was conducted to assess the impact of varying raw material and product prices on both PBT and NPV, based on an 89,000 MT/year capacity. Equations 8 and 9 show the PBT and NPV equations used to assess the profitability of the simulated CPP. The NPV is a financial concept used to forecast the present value of future cash flows, based on a series of cash flows and a specific discount rate, and it helps investors evaluate the profitability and viability of various investments.^41,42^ The NPV for the cumene production facility was estimated at 7% interest.

7 TCI=TDC+TIC+CFC

8 $$PBT=\frac{initial\;investment}{annual\;cash\;flow}$$

9 $$NPV=\sum_{t=1}^{n}\frac{C_{t}}{{\left(1+r\right)}^{t}}$$

where *C*_*t*_ is the net cash flow in each year; *r* is the interest rate; *t* is the year number; and *n* is the total number of years of the project.

Techno-economic data for the CPP were estimated by using the purchasing prices of raw materials and the selling prices of products reported across various markets. USD was preferred as the currency for the simulation program. The purchasing prices of benzene, propylene, and natural gas were set at 770 USD/MT,^43^ 966 USD/MT,^44^ and 3.50 USD/MMBtu,^45^ while the selling prices of cumene and DIPB were at 1075 USD/MT^16^ and 3108 USD/MT,^46,47^ respectively.

## 3 Results and Discussion

### 3.1 Description of the CPP

In the CPP, a steady-state petrochemical process, benzene and propylene are pressurized to reaction pressure using pumps and then heated to the required reaction temperature using flue gas from the combustion chamber. The heated feed mixture is introduced into a reactor operating at 350 °C and 30.75 bar, where benzene and propylene are converted into cumene and DIPB. The reactor effluent is subsequently cooled to enable gas–liquid separation. The gaseous components are separated from the liquid phase in a flash. The liquid stream is then sent to distillation columns, where benzene, cumene, and DIPB are separated based on differences in their boiling points to achieve high purity. Unreacted benzene is recycled back into the process via a benzene recycling line. Finally, the purified products, cumene, and DIPB are stored in storage tanks.^18,19,23^ This process was modeled using SPD for an annual cumene production capacity of 89,000 MT and then also simulated 29 times for TEA, based on various raw material/product prices. The flow rates and operating parameters for each process in the CPP with a capacity of 89,000 MT/year are described in detail below. Their operating conditions and flow properties are shown in Table 2, and they are close to the literature data.^23^

**Table 2:** Flow Properties of All Materials at the Inlet and Outlet of Each Equipment in the CPP and Their Operating Conditions^a^

stream names	pressure (bar)	temperature (°C)	vapor fraction	benzene (kmol/h)	propylene (kmol/h)	propane (kmol/h)	cumene (kmol/h)	DIPB (kmol/h)	total molar flow rate (kmol/h)	total mass flow rate (MT/h)
propylene	11.66	25	0	0	105	5.27	0	0	110.27	4.65
benzene	1.013	25	0	105	0	0	0	0	105	8.20
S-101	1.013	41.2	0	205.01	0.88	∼0.00	0.98	0	206.88	16.17
S-102	31.25	41.9	0	205.01	0.88	∼0.00	0.98	0	206.88	16.17
S-103	31.25	25.5	0	0	105	5.27	0	0	110.27	4.65
S-104	31.25	36.9	0	205.01	105.88	5.27	0.98	0	317.15	20.82
S-107	30.75	350	1	205.01	105.88	5.27	0.98	0	317.15	20.82
S-108	30.75	350	1	108.66	6.75	5.27	94.56	2.78	218.02	20.82
S-109	30.75	90	0.09	108.66	6.75	5.27	94.56	2.78	218.02	20.82
fuel gas	1.75	90	1	7.83	5.87	5.27	0.77	0	19.74	1.18
S-110	1.75	90	0	100.83	0.88	∼0.00	93.79	2.78	198.28	19.64
benzene recycling	1.75	57	0	100.01	0.88	∼0.00	0.98	0	101.88	7.97
S-111	1.75	179	0	0.82	0	0	92.81	2.78	96.4	11.67
S-112	2.10	178	0	0.82	0	0	92.71	0.03	93.56	11.21
S-113	2.10	45	0	0.82	0	0	92.71	0.03	93.56	11.21
S-114	2.10	222	0	0	0	0	0.09	2.75	2.84	0.46
S-115	2.10	45	0	0	0	0	0.09	2.75	2.84	0.46
cumene product	1.013	45	0	0.82	0	0	92.71	0.03	93.56	11.21
DIPB	1.013	45	0	0	0	0	0.09	2.75	2.84	0.46

stream names	pressure (bar)	temperature (°C)	O_2_ (kmol/h)	butane (kmol/h)	CO_2_ (kmol/h)	ethane (kmol/h)	methane (kmol/h)	N_2_ (kmol/h)	propane (kmol/h)	total mass flow rate (MT/h)
natural gas	3	25	0	0.05	0.20	0.30	31.50	0.30	0.12	0.54
air	3	25	117.9	0	0	0	0	443.63	0	16.20
S-105	3	25	117.9	0.05	0.20	0.30	31.50	443.93	0.12	16.74
S-106	3	1316.50	52.92	0.00	32.86	0.00	0.00	443.93	0.00	16.74
waste flue gas	2.5	365.20	52.92	0	32.86	0	0	443.93	0	16.74

a As a result of the combustion reaction, the waste flue gas also involves 64.63 kmol of water vapor.

#### 3.1.1 Benzene Pretreatment Way

All materials at 1.75 bar, 57 °C^18,23^ (the condensation temperature of benzene in the benzene column) with a molar flow rate of 101.88 kmol/h coming out from the benzene recycling line and benzene at 1.013 bar, 25 °C with a molar flow rate of 105 kmol/h coming out from the fresh benzene line are fed into the storage tank. After these materials are mixed in the benzene storage tank, they are fed at a molar flow rate of 206.88 from the benzene storage tank, operated at 1.013 bar and 41.2 °C, to 1. pump. The pressure of these organic materials in the liquid phase is raised from 1.013 to 31.25 bar using 1. pump.^23^ It also helps adjust the pressure during a gas-phase reaction in a reactor. The operating conditions/design specifications of the organic liquids leaving the benzene storage tank, along with their molar flow rates, are illustrated in Tables 2 and 3.

**Table 3:** Molar Flow Properties of All Materials in the Outlet of Three Storage Tanks and the Operating Parameters for the Tanks

component	benzene storage tank molar flow rate (kmol/h)	the cumene storage tank molar flow rate (kmol/h)	the DIPB storage tank molar flow rate (kmol/h)
benzene	205.01	0.82	0
cumene	0.98	92.71	0.09
DIPB	0	0.03	2.75
propane	∼0	0	0
propylene	0.88	0	0

operating parameters	value		
pressure (bar)	1.013		
exit temperature (°C)	41.2	45	45
residence time (h)	1		
working/vessel volume ratio (m^3^)	20.91	14.79	0.59

#### 3.1.2 Propylene Pretreatment Way

Propylene and propane at 11.66 bar and 25 °C, leaving the fresh propylene line, are fed at molar flow rates of 105 kmol/h and 5.27 kmol/h into 2. pump, respectively. The pressure of this mixture in the liquid phase is raised from 11.66 to 31.25 bar using 2. pump.^23^ It also helps adjust the pressure of the gas-phase reaction performed in the reactor, just as 1. pump adjusts the reactor pressure. On the other hand, the organic liquid mixture with 317.15 kmol/h leaving 1. and 2. pumps is transferred to the heat exchanger through 2. mixing. The molar flow rates of these organic liquids leaving 2. mixing are illustrated in Table 2.

#### 3.1.3 Heating Process

These organic liquids at 36.9 °C, leaving 2. mixing, are heated up to the gas-phase reaction temperature in the reactor via a multitube heat exchanger operated in countercurrent mode. Heat is transferred from the flue gas to the organic liquid mixture, raising its temperature from 36.9 to 350 °C.^23^ Consequently, the organic liquid mixture vaporizes and is fed to the reactor at 350 °C. The heat duty transferred from the flue gas to these liquids in the heat exchanger was calculated to be 5460.94 kW, which is close to 5327.8 kW reported in the literature.^23^ The flow properties of the flue gases at the inlet and outlet of the heat exchanger, as well as the operating parameters for the heat exchanger, are illustrated in Table 4.

**Table 4:** Mass Flow Rates of Flue Gas and Operating Parameters for the Heat Exchanger

operating parameters	entrance	exit
pressure (bar)	3	2.5
temperature (°C)	1316.50	365.20
flow rate of flue gases (kg/h)	16,739.97	
temperature decrease for the hot stream (°C)	951.30	
temperature increase for the cold stream (°C)	313.10	
outlet temperature of the hot stream (°C)	365.20	
outlet temperature of the cold stream (°C)	350	
heat transfer coefficient (W/m^2^·°C)	100	
pressure drop for tube stream (bar)	0.5	
pressure drop for shell stream (bar)	0.5	

#### 3.1.4 Gas-Phase Reactions for Cumene Production

The gas-phase reaction occurred between benzene and propylene at 30.75 bar and 350 °C, producing cumene as the main product and DIPB as a byproduct. The reaction mechanisms and their heats of reaction are shown in eqs 1 and 2. The reactor design for gas-phase reactions was simulated without requiring kinetic data, as SPD enables the design of various reactor types operated in continuous stoichiometric reaction mode. Design data, such as reaction conversion, residence time, and the working-to-vessel volume ratio, reported in the literature, are entered by the user rather than kinetic data. This mode also enables adjustment of the molar flow rates of all materials at the reactor inlet and outlet.^13,48^ The reactor modeled in this study was simulated based on a 6.50 m^3^ of the reactor volume and the molar flow rates at the reactor inlet and outlet reported by Turton et al. (2012).^23^ The molar flow rates of the materials around the reactor and the reactor design parameters simulated using SPD are presented in Table 5.

**Table 5:** Molar Flow Rates and Compositions of All Materials around the Reactor and Its Design Parameters

	inlet		outlet	
component	flow rate (kmol/h)	molar fraction (%)	flow rate (kmol/h)	molar fraction (%)
benzene	205.01	64.64	108.66	49.84
propylene	105.88	33.39	6.75	3.10
cumene	0.98	0.31	94.56	43.37
propane	5.28	1.66	5.28	2.42
DIPB	0	0	2.78	1.27
total molar flow rate	317.50		218.02	

design parameters	
length	9.388 m
diameter	0.939 m
volume	6.50 m^3^
working volume	5.85 m^3^
cooling duty	2715.63 kW
cooling rate	129.89 kg/s

The first reaction, which occurs between benzene and propylene in the gas phase, and the second reaction, which takes place between cumene and propylene produced in the reaction medium, are irreversible and exothermic (eqs 1 and 2). The activation energy of the first reaction is higher than that of the second reaction, and the gas-phase reaction at lower temperatures enhances the selectivity of cumene.^19^ Moreover, lower reaction temperature results in lower heat release from the exothermic reaction. The heat generated by the exothermic reactions in eqs 1 and 2 is removed from the reaction medium using cooling water to maintain the reaction temperature at 350 °C. The heat duty calculated by simulation was 2715.63 kW, which is consistent with the data (2733.33 kW (9840 MJ/h)) reported in the literature.^23^

#### 3.1.5 Cooling Process

Propylene and propane at 30.75 bar and 90 °C are in the gas phase, while benzene, cumene, and DIPB at the same conditions are in the liquid phase;^23^ thereby, the temperature of the materials at the reactor outlet should be reduced to 90 °C^19^ to enable gas–liquid separation. As a result, after the product, byproduct, and unreacted raw materials with high energy leave the reactor at the end of the gas-phase reaction at 350 °C and 30.75 bar, they are fed into a cooler to cool them from 350 to 90 °C. The molar flow rates of all materials around 1. cooling in Figure 1 are shown in Table 2. Moreover, the heating duty transferred from the gases at 350 °C to the cooling water, as calculated by the simulator, was 4918.21 kW. This value is close to 4555 kW (16,400 MJ/h) and 4822 kW (17.36 GJ/h) reported in previous studies.^18,23^

#### 3.1.6 Gas Separation Way

The gas–liquid mixture at 30.75 bar and 90 °C, leaving 1. cooling, is fed to the flash for gas–liquid separation to produce high-purity gases. Propylene and propane, due to their lower boiling points (Table 6) compared to benzene, cumene, and DIPB, leave the column at the top of the flash, while benzene, cumene, and DIPB, with their higher boiling points (Table 6), discharge the column at the bottom of the flash, which is operated at 1.75 bar and 90 °C.^19^ When comparing the boiling points of benzene, cumene, and DIPB, benzene has the lowest, while DIPB has the highest (Table 6). Therefore, a small amount of benzene and cumene was ensured to be discharged upstream of the column outlet, as liquids can vaporize even at temperatures below their boiling points. On the other hand, due to the solubility of gases in liquids, some propylene and propane were also allowed to pass through the downstream outlet of the column, along with the liquids. The heat is generated by burning the fuel gases leaving from the upstream of the flash in Figure 1, and it can be used to heat many parts of the production facility. For the flash simulation, the molar fractions in the streams leaving the column were taken from a study reported in the literature.^23^ The molar flow rates at the inlet and up-/downstream of the flash in Figure 1, as well as the fractions of gases, are illustrated in Table 6.

**Table 6:** Molar Flow Rates of All Materials around the Flash and the Fractions of Gases in the Flash

			molar flow rate (kmol/h)		
component	boiling point (°C)	vapor fraction (%)	inlet stream	upstream	downstream
benzene	80.09	7.21	108.66	7.83	100.83
cumene	152.45	0.81	94.56	0.77	93.79
DIPB	210	0	2.78	0	2.78
propane	–42.05	99.90	5.28	5.27	0.01
propylene	–47.65	87	6.75	5.87	0.88

#### 3.1.7 Benzene/Cumene/DIPB Separation Way

The boiling point difference is used to separate a liquid in an organic liquid mixture (benzene, cumene, and DIPB), yielding compounds of high purity. As a result, the organic liquid mixture from the flash is first fed to the benzene column and then to the cumene column.^17^ The benzene and cumene columns were operated at 1.75 and 2.1 bar, respectively.^23^ Because benzene is the most volatile compound in this liquid mixture, benzene leaves the upstream of the benzene column along with a small amount of cumene. The less volatile cumene and DIPB, along with the small amount of benzene, discharge from the downstream of the benzene column and enter the cumene column.^17,18^ Again, due to the boiling point difference, cumene is removed from DIPB in the cumene column. Cumene, with higher volatility than DIPB, leaves the upstream of the cumene column, while DIPB discharges from the downstream of the column. Cumene and DIPB are stored in packaging tanks. It is observed that cumene and DIPB are obtained with purities of 99.39% and 97.56% (mass), respectively, consistent with the values reported in the literature.^15^ The operating parameters for the benzene and cumene columns and the molar rates of all materials at the inlet and outlet of both columns are shown in Table 7.

**Table 7:** Molar Fractions of All Materials for the Benzene and Cumene Columns and the Operating Parameters of Both Columns

	benzene column			cumene column		
component	inlet stream to column molar fraction (%)	upstream of the column molar fraction (%)	downstream of the column molar fraction (%)	inlet stream to column molar fraction (%)	upstream of the column molar fraction (%)	downstream of the column molar fraction (%)
benzene	50.85	98.17	0.85	0.85	0.87	0
cumene	47.30	0.97	96.27	96.27	99.10	0.09
DIPB	1.40	0	2.88	2.88	0.03	2.75
propane	∼0.00	∼0.01	0	0	0	0
propylene	0.44	0.86	0	0	0	0
total molar flow rate (kmol/h)	198.28	101.88	96.40	96.40	93.56	2.84

	benzene column		cumene column	
component	relative volatility	percent (%) in distillate	relative volatility	percent (%) in distillate
benzene	8.31	99.19	18.58	100
cumene	1	1.05	3.99	99.90
DIPB	0	0	1	1
propane	176.93	100	0	0
propylene	222.84	100	0	0

operating parameter	benzene column	cumene column
reflux ratio	0.44	0.64
*R*/*R*_min_	1.314	1.92
feed quality (%)	70	70
column pressure (bar)	1.75	2.1
vapor line velocity (m/s)	3	3
number of theoretical stages	11.894	16.238
stage efficiency (%)	50	50

#### 3.1.8 Benzene Recycling Line

The fresh benzene feed and benzene at 1.75 bar and 57 °C,^18^ leaving the benzene column as a condensed liquid, are fed into the benzene storage tank, which is operated at 1.013 bar and 41.20 °C.^23^ The molar flow rates of all materials in the benzene recycle line are shown in Table 2.

#### 3.1.9 Natural Gas Combustion Process

The heating for the CPP is provided by the combustion of natural gas with air. Figure 1 shows the combustion of natural gas with air within a combustion chamber. In this combustion process, natural gas is stoichiometrically burned with 60% of excess air to ensure complete combustion in an air medium. After natural gas is burned in air, the flue gas temperature, as calculated by SPD, is 1316.5 °C. This value is sufficient to raise the temperature of all organic materials (leaving the second mixing) from 36.9 to 350 °C, which is close to the temperature (1400 °C) reported in the literature for burning natural gas with air.^36^ The flue gas at 1316.5 °C leaving the combustion chamber is fed to the heat exchanger, and benzene, propylene, and a small amount of cumene leaving 2. mixing are heated in this heat exchanger to raise their temperature to the reaction temperature. The heat transferred from the flue gas to raise the temperature of the organic liquid mixture fed to the heat exchanger from 36.9 °C to the reaction temperature of 350 °C was calculated to be 5460.94 kW. This value is consistent with 5327.8 kW and 5388.7 kW reported in the literature.^18,23^ The flow properties of the flue gases at the inlet and outlet of the heat exchanger, as well as the operating parameters for the heat exchanger, are illustrated in Tables 2 and 4.

### 3.2 CCD for the Optimization of PBT and NPV

#### 3.2.1 ANOVA Results

A full factorial design would require 81 (3^4^) experiments to optimize four parameters at three levels each. This high number of experiments was reduced to 29 using a CCD with five center points.^49^ Statistical models based on limited experiments were developed to link trends in PBT and NPV to raw material and product prices. To estimate the impact of raw material purchase prices and product selling prices on PBT and NPV, optimization data were derived by entering the 29 runs presented in Table 1 into the CCD program. This optimization program suggests a reduced cubic response surface and a 2FI (two-factor interaction) model to estimate PBT and NPV as functions of benzene price (*A*), propylene price (*B*), cumene price (*C*), and DIPB price (*D*). A thorough statistical analysis (ANOVA, diagnostic testing for model fit, and coefficient interpretation) has been performed to evaluate the model’s validity, structural characteristics, and predictive capacity.

The reduced cubic response surface model explains almost all the variation in PBT, captures complex interactions among the predictor variables, and is statistically robust. Table 8 shows that the ANOVA model is significant, with an *F*-value of 163.45 and a corresponding *p*-value of less than 0.0001.^50^ On the other hand, the 2FI model provides a highly effective, statistically rigorous framework for assessing the effects of raw-material and product prices on NPV. Table 9 shows that the ANOVA model is highly significant, with an *F*-value of 16,063.16 and a corresponding *p*-value of less than 0.0001.^50^ To estimate responses-1 and -2 in the experiment, PRESS is used; lower PRESS values^51^ indicate that the model is successful in predicting PBT and NPV and provides reliable results (Table 10). The results successfully explain a substantial portion of the variation in the PBT and NPV, making them suitable for analytical and predictive purposes.

**Table 8:** ANOVA Results for a Reduced Cubic Response Surface Model

source	sum of squares	d*f*	mean square	*F* value	*p*-value (prob > *F*)	
model	85.64	19	4.51	163.45	<0.0001	significant
*A*—benzene price	2.74	1	2.74	99.29	<0.0001	
*B*—propylene price	1.69	1	1.69	61.39	<0.0001	
*C*—cumene price	17.11	1	17.11	620.54	<0.0001	
*D*—DIPB price	1.12	1	1.12	40.54	0.0001	
*AB*	1.80	1	1.80	65.12	<0.0001	
*AC*	4.45	1	4.45	161.46	<0.0001	
*AD*	0.26	1	0.26	9.43	0.0133	
*BC*	3.37	1	3.37	122.11	<0.0001	
*BD*	0.21	1	0.21	7.51	0.0228	
*CD*	0.47	1	0.47	17.02	0.0026	
*A* ^2^	0.29	1	0.29	10.65	0.0098	
*B* ^2^	0.13	1	0.13	4.71	0.0581	
*C* ^2^	5.77	1	5.77	209.38	<0.0001	
*ABC*	1.12	1	1.12	40.75	0.0001	
*ACD*	0.18	1	0.18	6.40	0.0323	
*BCD*	0.14	1	0.14	5.10	0.0503	
*A* ^2^ *B*	0.38	1	0.38	13.71	0.0049	
*A* ^2^ *C*	0.23	1	0.23	8.23	0.0185	
*AB* ^2^	0.37	1	0.37	13.33	0.0053	
residual	0.25	9	0.028			
lack of fit	0.25	5	0.050			
pure error	0.000	4	0.000			
cor total	85.88	28				

**Table 9:** ANOVA Results for 2FI Model

source	sum of squares	d*f*	mean square	*F* value	*p*-value (prob > *F*)	
model	8399.24	10	839.92	16,063.16	<0.0001	significant
*A*—benzene price	1687.72	1	1687.72	32,276.8	<0.0001	
*B*—propylene price	1146	1	1146	21,916.65	<0.0001	
*C*—cumene price	5441.45	1	5441.45	1.04 × 10^5^	<0.0001	
*D*—DIPB price	123.08	1	123.08	2353.76	<0.0001	
*AB*	0.33	1	0.33	6.31	0.0218	
*AC*	0.33	1	0.33	6.31	0.0218	
*AD*	4.80 × 10^–3^	1	4.80 × 10^–3^	0.092	0.7655	
*BC*	0.33	1	0.33	6.32	0.0217	
*BD*	4.87 × 10^–3^	1	4.87 × 10^–3^	0.093	0.7638	
*CD*	4.73 × 10^–3^	1	4.73 × 10^–3^	0.09	0.7671	
residual	0.94	18	0.052			
lack of fit	0.94	14	0.067			
pure error	0	4	0			
cor total	8400.18	28				

**Table 10:** Results of the Model Summary Statistics Test for PBT and NPV

expressions	response-1	response-2
std. dev.	0.17	0.23
mean	3.34	29.49
CV %	4.97	0.78
press	7.12	3.25
*R*-squared	0.9971	0.9999
adjusted *R*-squared	0.9910	0.9998
predicted *R*-squared	0.9171	0.9996
adequate precision	50.90	463.22

The main effects for *A*, *B*, and *C* have *p*-values below 0.0001, while that of *D* is slightly above 0.0001, verifying that each input price has a direct influence on PBT and that almost all model terms in the reduced cubic structure are statistically significant.^50^ Moreover, *AB*, *AC*, *AD*, *BC*, *BD*, and *CD*, which exhibit pairwise interactions, are also statistically significant. These interactions show that the influence of each input price depends either on its own level or on the levels of other factors. Additionally, several higher-order terms of *A* ^2^, *C* ^2^, *ABC*, *ACD*, *A* ^2^ *B*, *A* ^2^ *C*, and *AB* ^2^ are also significant, and they exhibit three-factor interactions and meaningful quadratic effects, revealing the system’s multidimensional and nonlinear character.^52^ PBT shifts in a complex manner in response to simultaneous changes in raw material prices, requiring an appropriate higher-order response surface model. On the other hand, the main effects for *A*, *B*, *C*, and *D* have *p*-values below 0.0001, confirming that each input price has a direct influence on NPV and that almost all model terms in the 2FI structure are statistically significant.^50^

The coded-factor regression coefficients enable interpretation of the underlying relationships between PBT and the price variables (eq 10). *C* has the most substantial effect among the variables, with a coefficient of −1.46. Increasing cumene prices significantly reduce PBT, which is desirable for short-term investment returns.^53^ The negative coefficient of *D* is −0.22, indicating a weaker effect than that of *C*. On the other hand, the high coefficient of *A* has a greater effect than that of *B*. The positive coefficients for *A* and *B* are also 0.59 and 0.46, respectively, indicating that higher raw material prices are associated with higher PBT, rendering the investment unfeasible.^13^ The binary effect terms for prices *A*, *B*, *C*, and *D* reveal a complex interaction. The negative values for *AC* and *BC* interactions indicate that the effects of *A* and *B*, separately, reverse when *C* is high. *A* positive *AB* value suggests that it amplifies the effect of raw material prices on PBT, creating a synergistic effect.^54^ Compared with quadratic terms such as *A* ^2^, *B* ^2^, and *C* ^2^, *C* ^2^ plays an essential role in shaping the curvature of the response surface due to its high positive coefficient. It shows greater sensitivity to the PBT value as the cumene price increases, indicating a nonlinear response pattern. *A* ^2^ and *B* ^2^ contribute less to it than *C* ^2^ due to their lower coefficients. On the other hand, the coded-factor regression coefficients provide an interpretation of the underlying relationships between NPV and the price variables (eq 11). *C*, the selling price of cumene, has the most significant positive coefficient (+15.06) among the prices, reflecting its main product revenue and making it the largest factor in increasing NPV. *A* and *B* have strong negative coefficients of −8.39 and −6.91, which are consistent with the petrochemical economic theory that profitability decreases as raw material prices rise.^55^ *D*, as a comparatively minor byproduct revenue, increases NPV with a positive secondary income but a weak contribution. The *AB*, *AC*, and *BC* coefficients are so low, and their interactions reveal that the dual volatilities of benzene with propylene, propylene with cumene, and benzene with cumene introduce synergistic effects not detectable from the main effects alone.^54^ The positive coefficient on *AB* suggests that simultaneous increases in *A* and *B* prices disproportionately amplify their negative impact on NPV, indicating covolatility effects in raw material markets. The negative coefficients of *AC* and *BC* indicate that NPV decreases resulting from benzene or propylene price increases are mitigated as cumene prices rise, due to value-added buffering effects.^54^ DIPB plays a minimal role in the synergistic pricing dynamics, as the coefficients for *AD*, *BD*, and *CD* are pretty low and statistically insignificant. When PBT and NPV are directly calculated from raw material and product prices using the full regression equations expressed in coded factors, these equations can serve as an effective analytical tool for optimizing raw material–product prices and for evaluating the techno-economic performance of the CPP in both industrial applications and academic research.

The model fit statistics indicate that both response surface models provide a high-quality fit. The values for the *R*-squared, the adjusted *R*-squared, and the predicted *R*-squared of a reduced cubic response surface model are 0.9971, 0.9910, and 0.9171, while they are 0.9999, 0.9998, and 0.9996 for those of the 2FI model (Table 10).^56^ Such high *R*-squared values indicate an almost perfect fit, as both models explain 99.71% and 99.99% of the variability in PBT and NPV, respectively.^56^ The high adjusted *R*-squared values indicate that the models maintain strong explanatory power while avoiding overfitting, confirming that the included terms contribute meaningfully to the predictive performance. Generally, a predicted *R*-squared value above 0.90 indicates excellent generalization capability and suggests that both models are suitable for predicting PBT and NPV across varying price scenarios.^57^ The adequate precision values for both the reduced cubic response surface model and the 2FI model are 50.899 and 463.217, respectively, which exceed the acceptable value of 4, indicating that the models produce trustworthy signals across the design extent.^57^ The standard deviations (std. dev.) of 0.17 and 0.23 and the low coefficients of variation of 4.97% and 0.78% for both models reveal their accuracy and stability, respectively.^58^

10 $$PBT=2.82+0.59A+0.46B-1.46C-0.22D+0.34AB-0.53AC-0.13AD-0.46BC-0.11BD+0.17CD+{0.10A}^{2}+{0.069B}^{2}+{0.46C}^{2}-0.27ABC+0.11ACD+0.094BCD+{0.27A}^{2}B+{0.21A}^{2}C+{0.26AB}^{2}$$

11 NPV=29.49−8.39A−6.91B+15.06C+2.26D+0.14AB−0.14AC−0.017AD−0.14BC−0.017BD+0.017CD

#### 3.2.2 Effects of Raw Material and Product Prices on PBT and NPV

The 3D contour plots show how pairwise variations in raw material and product prices influence PBT (Figure 2). Each graphic visualizes economic sensitivity and nonlinear interactions among price inputs, revealing the cost structure hierarchy and cross dependencies among raw materials and products used in the petrochemical industry.^54^ A strong relationship between the purchasing prices of benzene and propylene, used as raw materials in the CPP, significantly increases PBT as raw material prices rise. Both concave shapes on both axes indicate that the PBT nonlinearly increases as the raw material price increases, whereas the other prices remain constant.^13^ The concave curve on the benzene price axis indicates that PBT is more affected by benzene prices, as the concave curve on the benzene price axis is higher on the PBT axis (Figure 2a).

**Figure 2:**
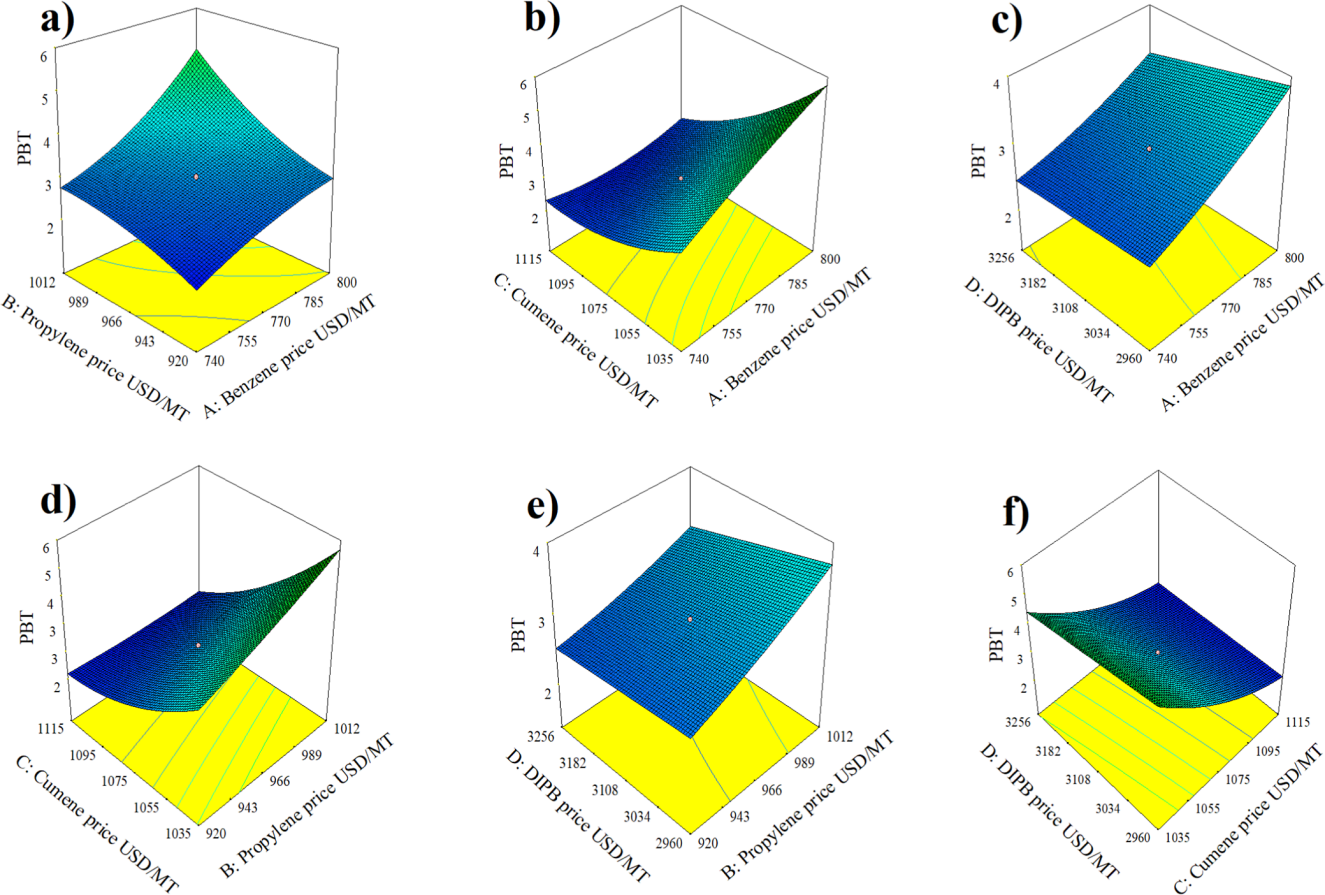
The 3D contour plot of the PBT results from CCD illustrating an interaction between (a) propylene-benzene prices, (b) cumene-benzene prices, (c) DIPB-benzene prices, (d) cumene-propylene prices, (e) DIPB-propylene prices, and (f) DIPB-cumene prices.

The 3D contour plots show how pairwise variations in raw material and product prices influence PBT (Figure 2). Each graphic visualizes economic sensitivity and nonlinear interactions among price inputs, revealing the cost structure hierarchy and cross dependencies among the raw materials and products used in the petrochemical industry. A strong relationship between the purchasing prices of benzene and propylene, used as raw materials in the CPP, significantly increases PBT as these prices rise.^59^ Both concave shapes on both axes indicate that the PBT nonlinearly increases as the raw material price increases, whereas the other prices remain constant.^13^ The concave curve on the benzene price axis indicates that PBT is more affected by benzene prices, as the concave curve on the benzene price axis is higher on the PBT axis (Figure 2a).

The profitability of the CPP depends on balancing the prices of cumene and benzene. The DIPB price has a negligible effect on PBT compared to the cumene price (Figure 2b,c). The decreasing DIPB price, with a much lower gradient, results in surface rises being very slight, showing that DIPB contributes marginally to the increase in PBT. Even at the lowest DIPB price, it exerts downward pressure, thereby suppressing the increase in PBT as benzene prices rise. DIPB revenues slightly enhance profitability and counterbalance the PBT increase driven by benzene price escalation (Figure 2c).

Figure 2d shows that increasing the cumene price significantly reduces PBT across the evaluated price range; even as propylene prices rise, the cumene price suppresses the increase in the PBT. On the other hand, PBT increases markedly at low cumene prices as the propylene price rises (Figure 2d). The surface in Figure 2e shows weaker overall gradients than those in Figure 2a–d, indicating a lower effect of DIPB and propylene prices on PBT. Both declining propylene prices and rising DIPB prices affect PBT only slightly. The nearly planar surface exhibits minimal nonlinear coupling among the variables. This 3D surface illustrates the relatively minor economic importance of DIPB and propylene compared to cumene and benzene (Figure 2e). Figure 2f shows that the cumene price has the most potent effect on PBT among all interactions. The surface rises sharply as the cumene price decreases, overshadowing the lower contribution of the DIPB price. At a constant cumene price, even as the DIPB price changes, the PBT changes very little, indicating that cumene prices overwhelmingly drive PBT and that DIPB provides a secondary economic contribution (Figure 2f).^13^

Figure 3 presents a series of 3D response surfaces showing the sensitivity of NPV to variations in raw material and product prices associated with the CPP. Each plot examines the interaction between two selected price variables while keeping the other prices constant. The six 3D surfaces in Figure 3 provide a comprehensive visualization of how NPV values change with price fluctuations. The dual effect of benzene and propylene price (raw materials) on the NPV is shown in Figure 3a. The 3D surface shows a gradient, illustrating that increases in both raw material prices lead to a systematic decrease in NPV, and this is an expected behavior as benzene and propylene prices constitute primary feedstocks in the OCs of the cumene facility, and their costs raise annual operating expenditures.^55^ The smoother surface inclination suggests that both raw materials significantly affect the NPV. The steeper slope along the benzene price axis indicates greater cost sensitivity compared with the propylene price axis (Figure 3a).

**Figure 3:**
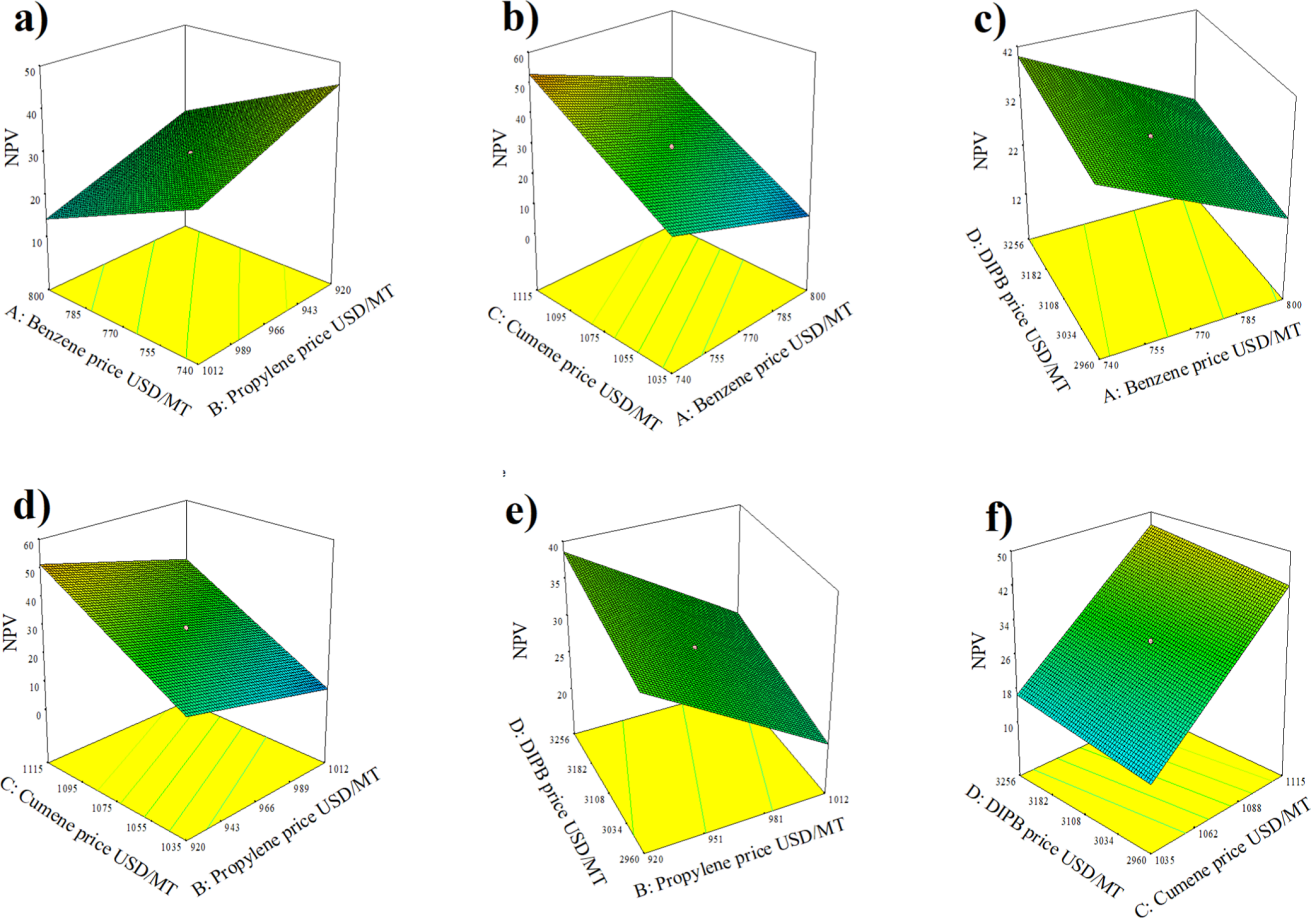
The 3D contour plot of the NPV results from CCD illustrating an interaction between (a) benzene-propylene prices, (b) cumene-benzene prices, (c) DIPB-benzene prices, (d) cumene-propylene prices, (e) DIPB-propylene prices, and (f) DIPB-cumene prices.

The interaction between cumene and benzene prices is illustrated in Figure 3b, and the 3D surface reveals a remarkable asymmetry, with NPV showing greater sensitivity to cumene price volatilities compared to that of benzene. The selling price of cumene directly and strongly affects revenue streams, as variations in the main product’s market revenue and price have pronounced effects on the profitability of the cumene facility. The downward slope in the cumene price axis is higher than that in the benzene price axis, indicating that NPV is more sensitive to the cumene price. The sharper decline along the cumene price axis underscores its greater role in assessing the facility’s economic viability.^60^ Also, opposite changes occur in both parameters, either increasing or decreasing the NPV (Figure 3b).

The interaction between DIPB and benzene prices is shown in Figure 3c. Unlike the 3D surfaces in Figure 3a,b, the 3D NPV surface illustrates a less steep gradient, suggesting that variations in DIPB price have a moderate effect on the profitability potential compared to raw materials. DIPB is produced as a byproduct in the cumene production facility, and its price contribution to NPV is low, but its profitability is notably high. The surface curvature implies a mild interaction effect: simultaneous increases in both prices lead to a compounded decrease in NPV, with the reduction being suppressed as the DIPB price increases. This suppression is less dramatic than in Figure 3a,b.

The 3D surface in Figure 3d captures the interaction between the cumene and propylene prices. The smooth planar surface reveals linear relationships within the evaluated price ranges for both materials. This surface illustrates a negative relationship between NPV and the propylene price and a strong positive relationship between NPV and the cumene price. The cumene price has a remarkable effect on increasing NPV, but the surface orientation indicates that this increase is also suppressed by the propylene price.

The NPV sensitivity to DIPB and propylene prices is shown in Figure 3e, and the NPV is strongly affected by the propylene price, whereas DIPB shows a more moderate influence. The interaction curves in the 3D surface show a clear slope, with propylene prices dominating, suggesting that economic volatility in propylene prices may disproportionately affect profitability. This result underscores the importance of risk management strategies for the procurement of propylene.

Finally, Figure 3f shows the dual effect between DIPB and cumene prices. The 3D NPV surface shows a more balanced trend between the two variables, indicating that DIPB and cumene are revenue-generating sources. The upward slope of the surface indicates that NPV increases significantly as the product prices reach their maximum, consistent with the typical behavior in product-price-driven profitability. This 3D surface shows the highest positive sensitivity, highlighting the central role of cumene sales in the facility’s revenue.

To help interpret the interaction effects more closely, the tendencies observed in the response surfaces are interpreted in terms of the cost and revenue components of the CPP. Among all interactions, the benzene–cumene interaction is the most dominant in affecting both the PBT and the NPV. This can be explained because benzene is the primary raw material and makes the largest contribution to OCs, whereas cumene is the main product and leads in revenue. Hence, any concurrent shifts in the prices of benzene and cumene would directly affect the financial equilibrium between expenses and revenue. In terms of economics, due to higher OCs resulting from the higher benzene price, PBT increases, while NPV decreases. Still, this negative impact can be significantly offset by rising cumene selling prices, as higher revenue from the product enhances cash flow and accelerates capital recovery. This accounts for the pronounced negative interaction coefficients (*AC*) in the regression equation and the steep slopes of the associated response surfaces. The curvature of the 3D RSM plots also reflects the nonlinear behavior of this association. At low cumene prices, the system becomes very sensitive to benzene price increases, and PBT rises sharply with a concomitant fall in NPV. At low cumene prices, the system is sensitive to raw material price changes; it exhibits a strong buffering effect at high cumene prices. In contrast, at higher cumene prices, the system is more resistant to raw material price volatility, thanks to its strong revenue-based buffering mechanisms. By contrast, DIPB-related interaction is quite weak, reflecting its nature as a byproduct and a minor source of total revenues. So too, propylene, although having a material effect on the cost of operations, has a financial impact that is secondary to that of benzene, given its smaller share of the total cost of raw materials. In sum, the response surfaces express an economic mechanism for profitability: the balance of two dominant cost drivers (benzene and propylene) and one primary source of revenue (cumene). This agreement between statistical results and the basic principles of petrochemical economics demonstrates the validity and physical meaning of the models established.

The data points align closely along a 45° line (the ideal identity line) for both PBT and NPV values, indicating a high linear correlation between actual and predicted PBT–NPV values and minimal systematic error (Figure 4).^61^ The various points for PBT and NPV values are evenly distributed across the region near the line. The reduced cubic response surface and 2FI models successfully uncover the underlying economic relationships between raw material–product prices and PBT. The high *R* ^2^ values for both models indicate their strong explanatory directions. Points clustered far from the line are absent from both graphs, and they do not exhibit curvature, indicating that the model’s functional form sufficiently captures the relationships between raw material–product prices and PBT–NPV and that no significant outliers or data inconsistencies are present.

**Figure 4:**
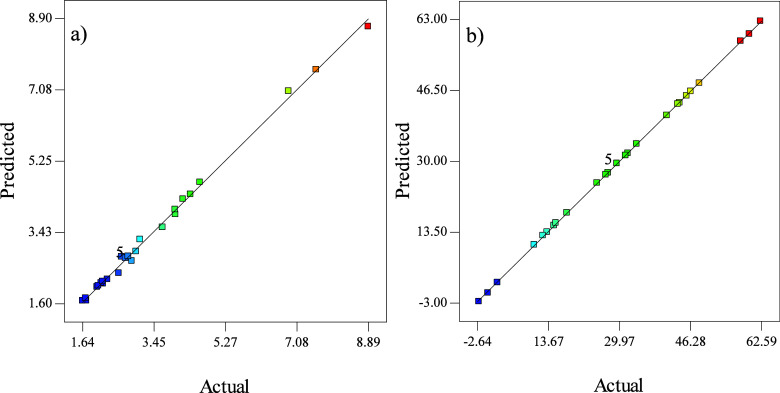
Predicted versus actual plot obtained by CCD based on (a) PBT and (b) NPV.

The scatter plots show that both models predicting PBT and NPV exhibit excellent accuracy, strong linearity, minimal bias, and robust performance across the entire price range. Both models capture the cost–profit relationships inherent in the petrochemical process, making them suitable for both diagnostic and predictive economic analyses. Both models are appropriate for price-sensitivity analysis in which volatility in benzene, cumene, propylene, or DIPB prices must be reliably translated into changes in PBT and NPV.

#### 3.2.3 Desirability for PBT and NPV

Table 11 highlights the importance of strategic procurement and price-risk management for maintaining profitability amid market price instability. Desirability was generated using a CCD based on the price ranges of the four variables, the minimum PBT value, and the maximum NPV value. The sensitivity analysis data in Table 11 illustrate how variations in benzene, propylene, cumene, and DIPB prices affect the process’s economic performance, as measured by PBT, NPV, and desirability. Across 30 studies, the facility’s most economically profitable potential occurs when benzene and propylene prices remain near their lower levels, strongly affecting the production costs of cumene and DIPB. All studies between 1 and 6 reveal minimal fluctuation in benzene and propylene prices and yield the highest NPVs and desirability points, indicating an economically profitable production facility under stable market conditions. As benzene and propylene prices rise, economic indicators begin to deteriorate, which is an expected outcome given the rise in feedstock costs. High propylene prices increase PBT but sharply reduce NPV, indicating that the process’s profitability is vulnerable to propylene price fluctuations. Moreover, studies 29 and 30, which reflect substantial increases in benzene and propylene prices, lead to reductions in desirability, dropping below 0.80 in the most unfavorable case. These results indicate that the process’s profitability is sensitive to volatility in raw material costs and that maintaining stable or reduced prices for key feedstocks is critical for economic sustainability.

**Table 11:** Sensitivity Analysis Derived by the CCD Program

study	benzene prices (USD/MT)	propylene prices (USD/MT)	cumene prices (USD/MT)	DIPB prices (USD/MT)	PBT (years)	NPV (million USD)	desirability
1	740.00	920.00	1114.93	3250.03	1.68	62.47	0.998
2	740.00	920.00	1114.15	3255.68	1.67	62.26	0.997
3	740.09	920.37	1115.00	3247.22	1.68	62.37	0.997
4	740.91	920.13	1114.88	3255.99	1.68	62.26	0.996
5	740.00	920.00	1115.00	3228.95	1.68	62.17	0.996
6	740.02	920.01	1115.00	3222.76	1.68	62.06	0.995
7	744.05	920.00	1115.00	3255.99	1.70	61.42	0.989
8	740.10	925.48	1115.00	3256.00	1.74	61.70	0.988
9	740.00	920.85	1115.00	3158.69	1.69	60.94	0.986
10	745.76	920.01	1115.00	3255.99	1.70	60.92	0.984
11	740.48	920.00	1115.00	3119.10	1.68	60.31	0.981
12	740.05	920.00	1115.00	3109.63	1.68	60.28	0.981
13	740.18	920.00	1115.00	3102.07	1.68	60.13	0.980
14	740.02	920.00	1115.00	3084.33	1.68	59.90	0.978
15	740.00	920.00	1115.00	3067.72	1.68	59.64	0.976
16	740.00	920.00	1114.83	3046.30	1.68	59.24	0.973
17	740.01	920.00	1115.00	3015.76	1.68	58.83	0.970
18	740.00	920.00	1103.28	3256.00	1.61	58.09	0.966
19	740.00	920.00	1113.40	3020.08	1.66	58.29	0.966
20	740.00	936.91	1115.00	3255.97	1.86	59.94	0.966
21	741.68	920.00	1114.50	2960.01	1.69	57.28	0.957
22	753.92	926.41	1114.97	3256.00	1.77	57.55	0.953
23	740.00	930.69	1112.25	2960.00	1.79	55.24	0.934
24	740.00	930.07	1111.49	2960.00	1.78	55.05	0.933
25	769.29	920.00	1114.89	3248.30	1.86	53.95	0.919
26	758.33	945.28	1115.00	3255.99	1.80	53.36	0.917
27	765.52	920.00	1115.00	2960.01	1.87	50.59	0.890
28	740.03	969.14	1115.00	2960.00	2.10	50.28	0.872
29	776.52	962.10	1115.00	3256.00	1.79	45.57	0.851
30	755.44	1012.00	1115.00	2960.63	1.89	39.30	0.788

### 3.3 TEA of the CPP

The TEA of the CPP enables the prediction of TCI, OC, and the unit production cost of cumene for an 89,000 MT/year capacity. The sizes of the equipment used in the production facility were determined from the mass and energy balances calculated by the SPD simulator for an 89,000 MT/year capacity. The costs for each process in Figure 1 were forecast using equipment-purchasing costs from the simulation program’s database. Benzene, propylene, cumene, and DIPB prices were entered into the simulator at 770 USD/MT, 966 USD/MT, 1075 USD/MT, and 3108 USD/MT, respectively. Cumene and DIPB revenues were calculated from their selling prices, while the costs of benzene, propylene, and equipment were derived from their purchasing price.

The TCI of the CPP is predicted to be 9.88 million USD, considering a single production rate (89,000 MT/year), as seen in Table 12, which provides a breakdown of TCI and the various parameters used to predict all costs. As production rates and equipment sizes increase, the TCI becomes more costly.^12,13^ The predicted equipment sizes and costs for the cumene production facility are listed in Table 13. The effect of production capacity on TCI is not examined in this study. Fluctuations in raw material and product prices do not notably affect the TCI, as the TCI includes various facility establishment expenses rather than raw material costs, as shown in Table 12. To recover the investment in the production facility quickly, an acceptable PBT is set at 5 years or fewer.^13,62^ The PBT and NPV of the CPP are predicted to be 2.80 and 29.383 million USD, based on a cumene production rate (89,000 MT/year) (Figures 2 and 3).

**Table 12:** TCI Parameters and Annual OC for the CPP

items		million USD
equipment purchase cost (PC)	listed equipment cost	1.67
installation	equipment-specific	0.56
process piping	0.2 × PC	0.59
instrumentation	0.1 × PC	0.67
insulation	0.03 × PC	0.05
electricals	0.1 × PC	0.17
buildings	0.05 × PC	0.75
yard improvement	0.1 × PC	0.25
auxiliary facilities	0.2 × PC	0.66
TDC		5.37
engineering expenses	0.25 × TDC	1.34
construction expenses	0.35 × TDC	1.88
TIC		3.22
contractor’s fee	0.05(TDC + TIC)	0.43
contingency	0.1(TDC + TIC)	0.86
CFC		1.29
TCI		9.88

total variable production costs	MUSD	%
raw materials	84.60	87.75
labor-dependent	6.25	6.48
facility-dependent	1.86	1.93
laboratory/QC/QA	0.94	0.98
utilities	2.76	2.86
total	96.41	100

**Table 13:** Properties and Cost of Equipment Used in the CPP

equipment	properties	cost (USD)
cumene column	column volume = 3.52 m^3^	281,000
benzene column	column volume = 2.42 m^3^	248,000
1. pump	pump power = 21.18 kW	112,000
reactor	vessel volume = 6.50 m^3^	110,000
heat exchanger (shell and tube)	heat exchange area = 92.39 m^2^	100,000
1. cooling (plate and frame)	heat exchange area = 30.74 m^2^	96,000
benzene storage tank	vessel volume = 20.91 m^3^	70,000
2. pump	pump power = 6.72 kW	67,000
combustion chamber	size/capacity = 16,740 kg/h	65,000
2. cooling (plate and frame)	heat exchange area = 14.09 m^2^	60,000
cumene storage tank	vessel volume = 14.79 m^3^	59,000
DIPB storage tank	vessel volume = 0.59 m^3^	30,000
flash	vessel volume = 4.09 m^3^	27,000
3. cooling (plate and frame)	heat exchange area = 0.18 m^2^	12,000
unlisted equipment		334,000
total equipment cost		1,671,000

OC is estimated at 96.41 million USD, as shown in Table 12, which presents the total variable production costs. OC for raw materials, labor-dependent, facility-dependent, laboratory/QC/QA, and utilities are calculated by the simulator as 84.60, 6.25, 1.86, 0.94, and 2.76 million USD, respectively. Purchasing expenses for raw materials accounted for 87.76% of the annual OC. The other portion of the yearly OC comprises labor-dependent, utilities, laboratory/QC/QA, and facility-dependent expenses, which together account for 12.24% of the total (Table 12). Benzene accounted for the largest share (59.12%) of the total raw material cost, followed by propylene (39.96%) and natural gas (0.92%).

Cumene production cost is estimated at 1090 USD/MT, based on the raw material purchasing costs mentioned above, for an annual cumene production rate of 89,000 MT. The simulator predicts annual revenues of 94.99 and 11.08 million USD/year for cumene and DIPB, respectively. Revenues from DIPB are 124 USD/MT per unit of cumene production, and total revenues amount to 1192 USD/MT, including the cumene unit selling price (1075 USD/MT). The fact that total unit revenues exceed the unit selling price of the main product reveals the facility’s profitability potential.^13^

A comparison of numeric results for cumene production with previous work is presented in Table 14 to assess the validity of the economic outcome. The estimated values of TCI, OC, PBT, and cumene revenue are consistent with the literature, which reflects the stability of the presented simulation and economic model. Yet, some deviations have been noticed. Among them, the cost of capital and OCs are influenced by minor differences in process design, such as reactor design, separation sequences, heat integration methods, and so on. Also, material balances and economic results are directly affected by variations in molar flow rates and conversion rates. Moreover, economic parameters, such as the discount rate, plant life, and cost-estimating method, may differ across studies and contribute to discrepancies in TEA. For instance, the estimation of TCI may differ due to varying equipment sizing methods or cost correlations resulting from the similar process parameters in these two cases. Furthermore, simulation platforms and modeling assumptions may also introduce additional discrepancies between the studies. In our work, the simulation results were validated using well-established design and cost estimation methodologies, e.g., Turton et al. (2012),^23^ and the results obtained were within reasonable, literature-consistent values. In conclusion, although minor deviations between the data in this study and other publications were observed, they are well within the range of previously reported results, demonstrating the validity of the modeling approach and the TEA’s sensitivity in this work.

**Table 14:** Comparison of the Economic Evaluation of the CPP with Previous Studies

various processes	Luyben^19^	Maity et al.^20^	Flegiel et al.^15^	Zhai et al.^21^	Pathak et al.^17^	Norouzi et al.^22^	this study
production capacity (10^3^)	89.36 t/year	95.12 t/year	306.88 t/year	96.88 t/year	95.68 t/year	97.92 t/year	89 MT/year
TIC (10^6^ USD)	7.33	8.13	17.87	8.22	5.90	8.09	9.88
OC (10^6^ USD/year)	135.15	143.02	475.37	132.11	131.54	141.67	96.41
PBT (year)	3	3	3	3	3	3	2.80
cumene revenue (10^6^ USD/year)	147.70	157.24	507.32	160.47	158.19	158.98	106.07

The economic data for the CPP, estimated by using the SPD simulator, were quantitatively compared with those reported in the literature. In this study, the reasonable PBT is estimated at 2.8 years, whereas previous studies reported a 3 year PBT.^18,19^ In these studies reported between 2010 and 2015, the economic analysis of the CPP indicates TCI ranging from 5.90 to 8.22 million USD, at almost the same production rate.^18^ The TCI (9.88 million USD) in this study is higher than that of previous studies, which can be attributed to inflation increasing between 2015 and 2025 in the United States.^63^ On the other hand, OC was reported in previous studies^18^ to range from 131.54 to 143.02 million USD, which is higher than the 96.41 million USD reported in this study. As raw material costs are the largest contributor to OC, the purchasing prices (93.43 USD/kmol and 56.50 USD/kmol, respectively)^18^ of benzene and propylene reported in previous studies are considerably higher than those used in this study; thus, the SPD simulation predicts a lower OC. In a separate study that evaluated the same production rate and various design scenarios, the OC is close to that estimated in this study, ranging from 90.883 to 94.204 million USD.^19^ The unit production cost of cumene is estimated at 1090 USD/MT by SPD, which is close to the reported production costs of 1015–1025 USD/MT by Norouzi et al., depending on the different production scenarios.^22^ As a result, the economic data obtained from the TEA of CPP are generally consistent with those reported in the literature.

Figure 5 presents the full sensitivity analysis of NPV to economic and financial assumptions and provides important insights into the economic nature of cumene production. As seen in Figure 5a, the NPV is most sensitive to changes in the revenue and cost of raw materials. This is in line with the known TEA wisdom that feedstock costs and product revenues are the largest contributors to the overall profitability of petrochemical processes. OCs are dominated by benzene and propylene, the major feedstocks, so fluctuations in their prices have a direct and significant effect on the NPV. Similar trends have been widely reported in the petrochemical TEA, where the cost of raw materials is the primary economic driver.^12,13,23,64^ In contrast, utility, labor-related, and plant-related costs have relatively little influence on NPV, suggesting that these cost terms are minor components of total OCs. This trend was consistent with previous conclusions that utilities and labor are significant but are generally considered a second-order effect in process economics relative to feedstock prices.^12,13,23,39^ The approximately linear trends visible in Figure 5a indicate that the economic response is approximately linearly proportional to changes in the parameters within the selected variation range. Such linear sensitivity behaviors have also been observed in early-stage TEA (on account of very small perturbation windows to verify the economic robustness of the process).^64^ Figure 5b shows the impact of the discount rate on NPV with respect to different plant lifetimes. As anticipated, the NPV declines monotonically as the discount rate increases, reflecting a lower present value of the future cash flows. This inverse relationship is at the heart of discounted cash flow analysis and is well outlined in the literature for economic evaluation.^39^ However, a longer plant lifetime contributes to higher NPV since more cash inflow is obtained by running longer. The gap between the curves shows that project life is substantial in long-term investment decisions, particularly for energy-intensive industries such as petrochemicals. In conclusion, the sensitivities in this study demonstrate that the feasibility of producing cumene is primarily determined by the cost of feedstock and the market value of the product; financial factors also influence the investment appeal under different economic situations.

**Figure 5:**
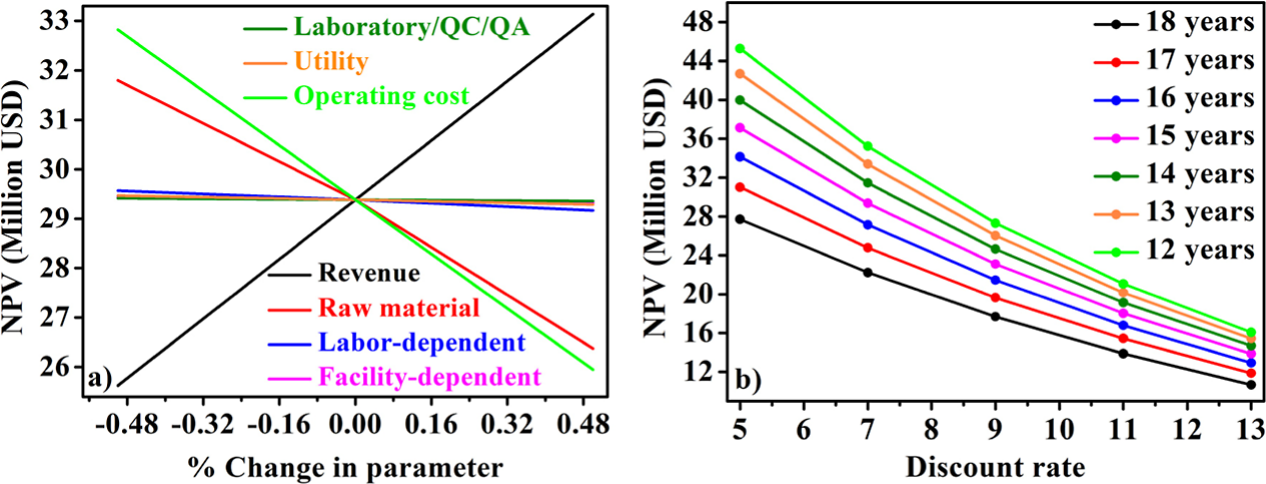
Economic sensitivity of the CPP: (a) key cost portions and revenue contributions for NPV and (b) discount rate influence on NPV at various plant lifetimes.

## 4 Conclusion

CCD successfully optimized PBT and NPV in the CPP economic analysis using data from the SPD program. When used alongside economic data generated by a simulation program, it provides a powerful tool for optimizing and describing data behavior, enabling the efficient identification of economic data correlations before investing in an industrial production process. All the variations in PBT and NPV directly affected by raw material and product prices are explained by both the reduced cubic response surface and 2FI models, respectively, and both models for ANOVA analysis are significant, with *F* values of 163.45 (*p*-value < 0.0001) and 16,063.16 (*p*-value < 0.0001). The *R*-squared, adjusted *R*-squared, and predicted *R*-squared for both models are well above 0.90, indicating excellent generalization and confirming their suitability for estimating PBT and NPV across varying price scenarios. Three-dimensional graphs derived from both models show that PBT rises notably as raw material prices increase and that NPV increases substantially as product prices rise, especially for cumene. The coded-factor regression coefficients derived from both ANOVA results allow the interpretation of the underlying relationships between economic outputs and the prices of the raw material and the product.

A detailed TEA of the CPP was modeled for an 89,000 MT/year of cumene production capacity, based on average material prices. The results reveal that the acceptable PBT and NPV are 2.8 years and 29.383 million USD, respectively, using the same price levels. The TCI and annual OC of the process are predicted to be 9.88 and 96.41 million USD, respectively, at the same production rate. The unit production cost of cumene is 1090 USD/MT based on the average prices of all materials. The simulated CPP for an 89,000 MT/year benzene production capability indicates that it is a feasible investment, a viable option, and an attractive choice. In light of the simultaneous use of SPD and CCD programs before investing in petrochemical production plants, future studies could focus on economic variables and optimize financial analysis predictions.

From a scale-up viewpoint, combining TEA with CCD optimization constitutes an attractive platform for industrial implementation. The results show that the economic performance of the process is most affected by variables dependent on the market, mainly raw material and product prices, which need to be closely monitored during scale-up. The sensitivity and 3D RSM analyses offer practical guidance for identifying optimal operating and economic conditions at the large scale. Such instruments allow for a more reliable decision by considering interaction effects among influential variables. Hence, the applied method can assist in the design and operation of cost-effective industrial-scale cumene production processes while accounting for the importance of economic flexibility in the face of volatile market conditions.

## Nomenclature

*A*: benzene price
kJ: kilojoule
*R*: reflux ratio
ANOVA: analysis of variance
kW: kilowatt
*R*_min_: minimum reflux ratio
*B*: propylene price
m: meter
RSM: response surface methodology
Bbl: a barrel of crude oil
M: million
*R* ^2^: coefficient of determination
*C*: cumene price
min: minute
s: second
CAGR: compound annual growth rate
MJ: megajoule
SPD: SuperPro Designer
CCD: central composite design
m^2^: meter square
std. dev.: standard deviation
CFC: contractor’s fee and contingency
m^3^: meter cubic
t: ton
COVID-19: coronavirus disease 2019
MMBtu: British thermal unit
TCI: total capital investment
CPP: cumene production process
MT: metric ton
TCIs: total capital investments
CV: coefficients of variation
NPV: net present value
TDC: total direct cost
*D*: DIPB price
NPVs: net present values
TEA: techno-economic analysis
DIPB: diisopropylbenzene
OC: operating cost
TIC: total indirect cost
eq: equation
OCs: operating costs
USA: United States of America
eqs: equations
*P*: probability value
USD: United States dollar
GJ: gigajoule
PBT: payback time
V9: version 9
h: hour
PC: equipment purchase cost
W: watt
Inc.: incorporated
QC: quality control
2FI: two-factor interaction
kg: kilogram
QA: quality assurance
°C: degree celsius
